# miR-616-5p Promotes Invasion and Migration of Bladder Cancer *via* Downregulating NR2C2 Expression

**DOI:** 10.3389/fonc.2021.762946

**Published:** 2021-12-09

**Authors:** Wenbiao Ren, Jiao Hu, Huihuang Li, Jinbo Chen, Jian Ding, Xiongbing Zu, Benyi Fan

**Affiliations:** ^1^ Department of Urology, Xiangya Hospital, Central South University, Changsha, China; ^2^ National Clinical Research Center for Geriatric Disorders, Xiangya Hospital, Central South University, Changsha, China

**Keywords:** miR-616-5p, NR2C2, bladder cancer, invasion, migration

## Abstract

**Background:**

MicroRNAs, small non-coding RNA molecules with about 22 nucleotides in length, play a significant role in the development of bladder cancer. Previous studies found that miR-616-5p could promote the progress of cancers. However, its role in bladder cancer remains unclear. In the study, we aimed to demonstrate how miR-616-5p impacts the invasion and migration of bladder cancer and its potential downstream targets.

**Methods:**

Firstly, qRT-PCR was used to detect the expression of miR-616-5p in normal bladder uroepithelial cell lines and bladder cancer cell lines. Then, chamber–transwell invasion and wound healing migration assays were used to detect the roles of miR-616-5p and NR2C2 in invasion and migration. Subsequently, Western blot was used to evaluate the regulation effects of miR-616-5p and NR2C2. Finally, luciferase assays were performed to manifest the mechanism of miR-616-5p and NR2C2 regulation.

**Results:**

We found that miR-616-5p was upregulated in bladder cancer, and it could promote the invasion and migration of bladder cancer *in vitro*. Moreover, we demonstrated that NR2C2 was a downstream target of miR-616-5p. miR-616-5p could inhibit the expression of NR2C2 by binding to the 3′UTR of NR2C2 mRNA. Importantly, patients with a high expression of NR2C2 showed better prognoses in bladder cancer.

**Conclusions:**

This study identifies that miR-616-5p can promote bladder cancer progression *via* altering the expression of NR2C2. Therefore, identifying miR-616-5p expression levels might be a useful strategy for developing potential therapeutic targets in bladder cancer.

## Introduction

Bladder cancer is one of the most common cancers in the world, especially in male patients (1, 2). Bladder cancers were classified as non-muscular invasive bladder cancer (Ta and T1) (NMIBC) and muscular invasive bladder cancer (T2–4) (MIBC) (3). About 75% were diagnosed as NMIBC, while the other 25% were MIBC (4). For patients with NMIBC, transurethral resection of the bladder tumor is the most commonly used treatment. However, patients with NMIBC have a risk of recurrence and progression rate, particularly in patients with TaT1 tumors and carcinoma *in situ* (CIS) (5). Radical cystectomy with bilateral lymphadenectomy is a standard surgery for MIBC, and platinum-based perioperative chemotherapy may be used in metastatic patients (6). Nevertheless, the prognosis of metastatic MIBC remains unfavorable with a 5-year overall survival rate of approximately 15% (7). Therefore, it is urgent to study molecular markers and therapeutic targets of bladder cancer to improve early diagnosis and treatment.

MicroRNAs (miRNAs) are a group of non-encoding RNAs with about 22 nucleotides in length (8). miRNAs are mainly involved in post-transcriptionally modulating the expression of target genes by inhibiting the translation of target mRNAs (9). miRNAs have been found to play an important role in the progress of various tumors, including bladder cancer (10, 11). miR-616-5p, a member of the miR-616 family, has been repeatedly reported to be upregulated in several cancers (12, 13). In ovarian cancer, miR-616-5p was upregulated by the downregulation of long noncoding RNA TUSC7, thus promoting cell growth, invasion, and migration through the GSK3β pathway (12). In lung cancer, silencing miR-616-5p markedly suppressed the migration and invasion of lung cancer cells *in vitro* and NSCLC metastasis *in vivo* (13). However, the expression status and function of miR-616-5p in bladder cancer remain unclear.

Nuclear receptor subfamily 2 group C member 2 (NR2C2), also known as testicular nuclear receptor 4 (TR4) plays an important role in maintaining physiological homeostasis (14). Recently, studies demonstrated that NR2C2 could act as an oncogene or tumor suppressor in various cancers (15–17). In papillary thyroid cancer, NR2C2 could promote invasion *via* activating circ-FNLA/miR-149-5p/MMP9 signaling (15). Similar results can also be found in clear cell renal cell carcinoma (16). Nevertheless, previous studies also found that NR2C2 could act as a tumor suppressor by modulating DNA damage/repair systems in prostate cancer (17). However, whether NR2C2 could correlate with miRNAs to impact bladder cancer progression remains unknown.

In this study, we found that miR-616-5p could promote the invasion and migration of bladder cancer cells *via* suppressing its potential downstream target NR2C2.

## Materials and Methods

### Cell Culture and Chemicals

Normal human urothelial epithelial cell line SV-HUC-1 and bladder cancer cell lines 5637, TCCSUP, J82, 647V, T24, UMUC3, and 293T cells were obtained from the American Type Culture Collection. SV-HUC-1 cells were cultured in Kaighn’s Modification of Ham’s F‐12 Media. All bladder cancer cells and 293T cells were cultured in Dulbecco’s modified Eagle’s medium. The culture media were supplemented with 10% fetal bovine serum, antibiotics (100 units/ml penicillin and 100 mg/ml streptomycin), and 2 mM glutamine (Invitrogen). The cells were cultured in an incubator with humidified 5% CO_2_ environment at 37°C.

### Lentivirus Packaging

293T cells were seeded and incubated until they reached a density of 40%. The medium was changed 4 h prior to transfection. Then, 20 μg target plasmid with 10 μg pMD2G envelope plasmid and 10 μg psAX2 packaging plasmid was co-transfected into 293T using the standard calcium phosphate transfection method. The lentivirus soup was collected twice after incubating for 48 or 72 h. The lentiviruses should be used immediately or frozen at −80°C for later use. The primers for plasmid construction were as follows: pLKO.1-miR-616-5p inhibitor sequence, forward: 5′- CCGGGTCAGCTCTTAGTATTCTAAAAGTCACTGAAGATCAGGTTTTGAGT-3′, reverse: 5′- ATGATATTACTCAAAACCTGATCTTCAGTGACTTTTAGAATACTAAGAGCTGAC -3′; pLKO.1-oemiR-616-5p sequence, forward: 5′- CCGGCTCAAAACCCTTCAGTGACTTGGATCCAAGTCACTGAAGGGTTTTGAGTTTTTTG -3′, reverse: 5′- AATTCAAAAAACTCAAAACCCTTCAGTGACTTGGATCCAAGTCACTGAAGGGTTTTGAGT -3′; pLKO.1-shNR2C2 sequence, forward: 5′-CCGGCCAGCACAAGCCAGATTGAAAGGATCCTTTCAATCTGGCTTGTGCTGGTTTTTG-3′, reverse: 5′-AATTCAAAAACCAGCACAAGCCAGATTGAAAGGATCCTTTCAATCTGGCTTGTGCTGG-3′; pWPI-oeNR2C2 sequence, forward: 5′-TTTCGACATTTAAATTTAATATGACCAGCCCCTCCCCACG-3′, reverse: 5′-ATTCCTGCAGCCCGTAGTTTCTATAGACTGGCTCCGGTGA-3′.

### Transwell Invasion Assay

Matrigel invasion assay was used to assess cell invasion capacity. Then, 100 μl of serum-free medium with Matrigel (BD Corning) (1:10) was plated in 8.0-μm-filter-membrane transwell chambers and incubated at room temperature overnight. Furthermore, 1 × 10^5^ cells suspended in 150 μl of serum-free medium were placed on the upper chamber. Then, 750 μl of culture medium with 10% serum was placed in the lower chamber. After incubating for 24 h, the invaded cells were fixed by methanol, dyed with 0.5% crystal violet, and observed under the microscope. Each group included triplicate results, and image J software was used to quantify the invaded cells.

### Wound Healing Assay

The cells were seeded into six-well tissue culture plates. After incubating, when the cells grew to a density of 90%, a sterile 200-μl tip was used to scratch the plate gently. Then, phosphate-buffered saline was used to wash the cells twice, and a fresh medium was replenished. Images were taken at 0 and 12 h after incubation. The wound width decreasing percentage was compared between each group.

### RNA Extraction and qRT-PCR Analysis

Trizol reagent (Invitrogen) was used to extract RNA, and the miRNAs were isolated using PureLink^®^ miRNA kit. First, 2 µg RNA was used for poly A polymerase by adding 0.4 μl poly A enzyme, 2 μl 5× RT buffer, 1 μl 10 mM ATP, and appropriate ddH_2_O to 10 μl in total at 37°C for 20 min. Second, the annealing step was conducted by adding 1 μl RT anchor primer (50 μM) to the poly A reaction and annealing at 65°C for 5 min and 4°C for 2 min. The last step was cDNA synthesis at 42°C for 60 min by adding 2 μl 10 mM dNTP, 2 μl 5× RT buffer, 1 μl RevertAid (Thermo Fisher), and appropriate ddH_2_O to a total of a 20-μl system. The qRT-PCR protocol was as follows: 95°C for 2 min, followed by 45 cycles at 95°C for 15 s, and 60°C for 45 s. U6 was used as a normalized control.

### Western Blot

T24 and UMUC3 cell lines were harvested and lysed by RIPA lysis buffer. Total protein was quantified using a bicinchoninic acid protein assay kit (Thermo Fisher). Then, 30 μg of protein samples were resolved by 10% SDS-PAGE gel (80 V, 120 min) and transferred to polyvinylidene difluoride membrane (105 V, 100 min). Then, the membranes were blocked with 5% pure milk for 1 h. The membranes were incubated with primary antibodies at 4°C overnight. On the next day, the blots were incubated with HRP-conjugated secondary antibodies for 1 h at room temperature. The bands were visualized with ECL system (Thermo Fisher). The primary antibody information for western blot is as follows: GAPDH (AC033, 1:1,000), NR2C2 (MA5-26855, 1:1,000), P53 (sc-126, 1:1,000), P27 (A16722, 1:1,000), CHK2 (sc-9064, 1:1,000), HOXA5 (sc-13199, 1:1,000), and RARB (A4535, 1:1,000).

### RNA Binding Protein Immunoprecipitation

The lysates from T24 and UMUC3 cells were incubated with the antiIgG or antiAGO2 antibody for 16 h at 4°C with agitation. Protein A/G-agarose beads were then added, and binding RNAs were eluted. The eluted RNAs were analyzed by qRT-PCR.

### Luciferase Assay

Six-hundred-base-pair fragments of NR2C2 3′UTR containing wild-type or mutant miRNA-responsive elements were cloned into the psiCHECK™-2 vector construct (Promega) downstream of Renilla luciferase ORF. The cells were seeded in 24-well plates. When the cells grew at a density of 60%, the plasmids were transfected with lipo3000 and p3000 (Invitrogen) according to the instructions of the manufacturer. Luciferase activity was measured by Dual-Luciferase Assay (Promega). The primers for plasmid construction were as follows: psiCHECK-2-NR2C2 3′UTR wild type, forward: 5′- CAGTAATTCTAGGCGATCGCATGCAGTAAGTGGGAGTGTGG -3′, reverse: 5′- AGATATTTTATTGCGGCCAGCGGTCAACAGGAAGAAATTGTCC-3′; psiCHECK-2-NR2C2 3′UTR mutant, forward: 5′- CAGTAATTCTAGGCGATCGCATGCAGTAAGTGGGAGTGTGG -3′, reverse: 5′- TCAAGAGTCATGTCTGCCCACCTTGCACTTTTTG -3′; forward: 5′- GTGGGCAGACATGACTCTTGACAGTACTTCCAATACAATTG -3′, reverse: 5′- AGATATTTTATTGCGGCCAGCGGTCAACAGGAAGAAATTGTCC-3′.

### Statistical Analysis

Data are shown as mean ± SD. SPSS 23 and GraphPad Prism 7.0 were used to analyze the statistics, including Student’s *t*-test, one-way ANOVA, and log-rank test. *P <*0.05 was considered a statistically significant difference.

## Results

### miR-616-5p Expression Is Higher in Bladder Cancer Than in Normal Bladder Tissues or Cells

**Figure 1:**
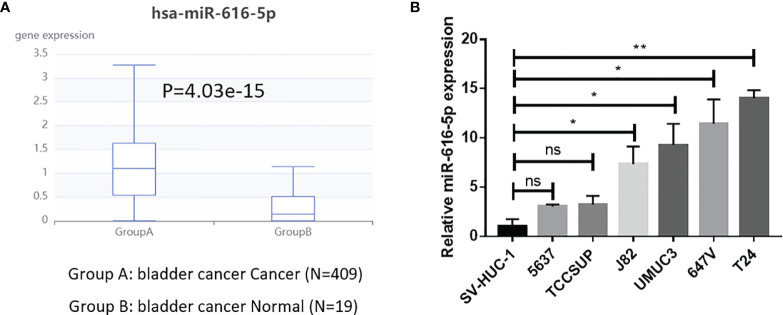
miR-616-5p expression is higher in bladder cancer than in normal bladder tissues or cells. **(A)** Expression of miR-616-5p in bladder cancer tissues and adjacent normal tissues based on The Cancer Genome Atlas database. **(B)** Expression of miR-616-5p in normal bladder uroepithelial cell line SV-HUC-1 and bladder cancer cell lines 5637, TCCSUP, J82, UMUC3, 647V, and T24. Quantification data are presented as mean ± SD, and significant differences are indicated by *P < 0.05, **P < 0.01 and ns, not significant compared to the controls.

First, we investigated the expression of miR-616-5p in patients with bladder cancer. An analysis of The Cancer Genome Atlas (TCGA) data demonstrated that the expression of miR-616-5p in bladder cancer tissues was higher than that in adjacent normal tissues (**Figure 1A**) (18). Similarly, as shown in **Figure 1B**, the expression of miR-616-5p in normal bladder uroepithelial cell line SV-HUC-1 was lower than that in bladder cancer cell line 5637, TCCSUP, J82, UMUC3, 647V, and T24. Taken together, **Figure 1** manifested that miR-616-5p might promote tumor genesis of bladder cancer.

### miR-616-5p Promotes Bladder Cancer Cell Invasion and Migration

**Figure 2:**
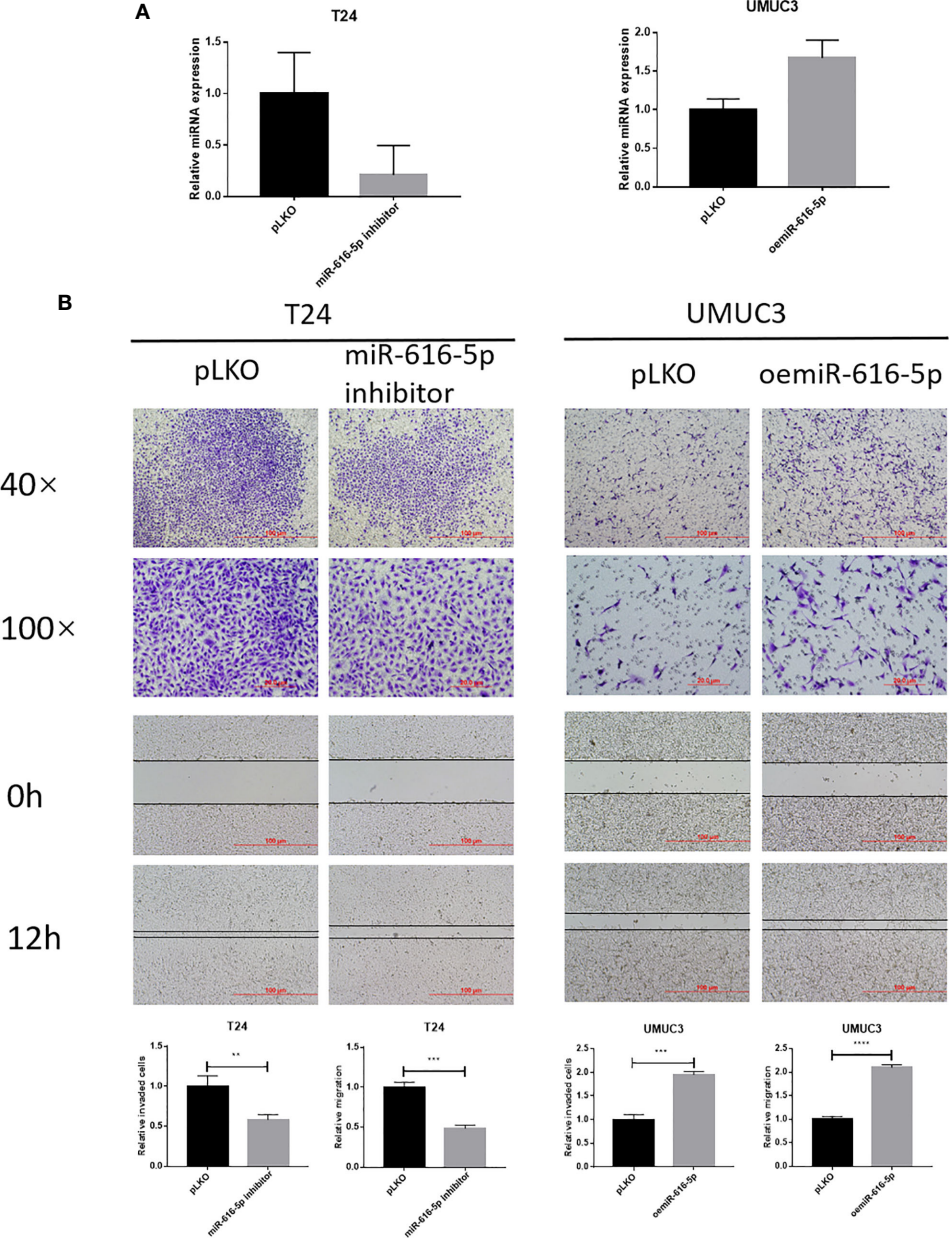
miR-616-5p promotes bladder cancer cell invasion and migration. **(A)** qRT-PCR assay for miR-616-5p expression in T24 cells transduced with vector control pLKO and miR-616-5p inhibitor and UMUC3 cells transduced with pLKO and overexpressed miR-616-5p. **(B)** Chamber–transwell invasion and wound healing migration assays performed in T24 cells transduced with pLKO and miR-616-5p inhibitor and UMUC3 cells transduced with pLKO and oemiR-616-5p. For **(B)**, quantification data are presented as mean ± SD, and significant differences are indicated by **P < 0.01, ***P < 0.001, and ****P < 0.0001 compared to the controls.

In this study, we used T24 and UMUC3 cell lines to study the biological function of miR-616-5p in bladder cancer. First, we construct the inhibited and overexpressed miR-616-5p plasmid with the pLKO vector. Then, 293Tcells were used to package the virus. Next, the miR-616-5p inhibitor virus was transduced into T24 cells, and the oemiR-616-5p virus was transduced into UMUC3 cells. The qRT-PCR result showed that the efficiency of the overexpression and inhibition of miR-616-5p was very good (**Figure 2A**). Then, we did experiments to explore the effect of miR-616-5p on phenotype. Chamber–transwell invasion and wound healing migration assays were performed in T24 and UMUC3 cells. As shown in **Figure 2B**, when we inhibited the miR-616-5p expression, the invasion and migration of T24 cells decreased. The invasion and migration increased when miR-616-5p was overexpressed (**Figure 2B**). In sum, **Figure 2** illustrated that miR-616-5p could promote invasion and migration in multiple bladder cancer cell lines.

### NRC2C2 Is Negatively Correlated With miR-616-5p and Inhibits Bladder Cancer Cell Invasion and Migration

**Figure 3:**
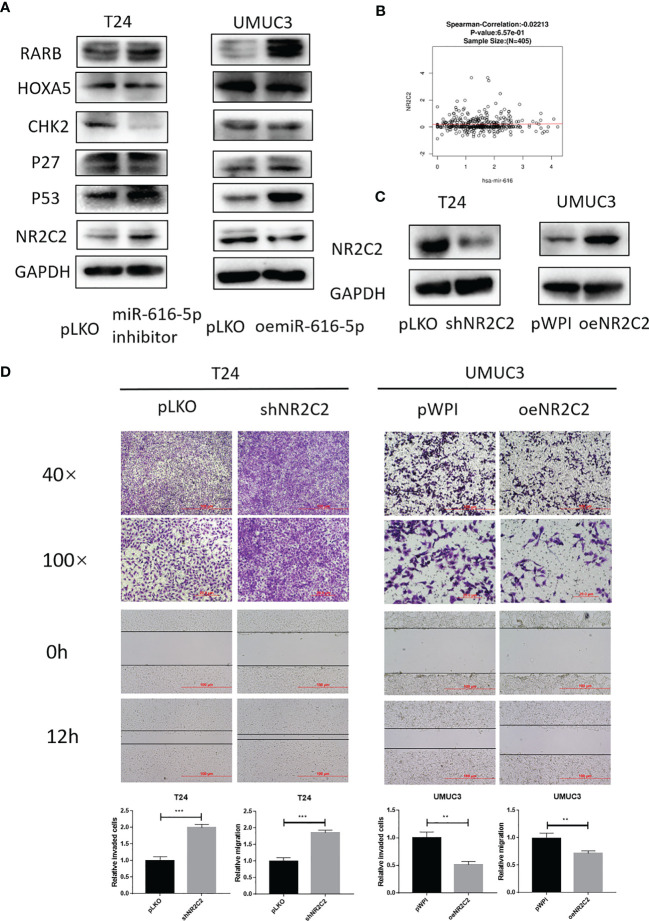
NR2C2 is negatively correlated with miR-616-5p and inhibits bladder cancer cell invasion and migration. **(A)** Western blot assay for screening the downstream target genes in T24 cells transduced with pLKO and miR-616-5p inhibitor and UMUC3 cells transduced with pLKO and oemiR-616-5p. **(B)** Correlation between miR-616 and NR2C2 expression in bladder cancer according to LinkedOmics database. **(C)** Western blot assay for NR2C2 expression in T24 cells transduced with pLKO and shNR2C2 and UMUC3 cells transduced with pWPI and oeNR2C2. **(D)** Chamber–transwell invasion and wound healing migration assays performed in T24 cells transduced with pLKO and shNR2C2 and UMUC3 cells transduced with pWPI and oeNR2C2. For **(C)**, quantification data are presented as mean ± SD, and significant differences are indicated by **P < 0.01, and ***P < 0.001 compared to the controls.

To find out the potential downstream target of miR-616-5p, we screened several cancer-related genes with Western blot (WB) assay. As shown in **Figure 3A**, only NR2C2 could be negatively regulated by miR-616-5p. Moreover, we found that the expression of miR-616 and NR2C2 was negatively correlated in bladder cancer according to the LinkedOmics database (**Figure 3B**) (19). Next, we constructed the overexpression and knock-down plasmids of NR2C2. The WB assay showed that the efficacy of shNR2C2 and oeNR2C2 was very good (**Figure 3C**). Then, invasion and migration assays were conducted to determine the effect of NR2C2 on phenotype. As we can see from **Figure 3D**, shNR2C2 could increase invasion and migration in T24 cells, and oeNR2C2 could decrease invasion and migration in UMUC3 cells. **Figure 3** altogether revealed that miR-616-5p could downregulate NR2C2 expression, and NR2C2 could inhibit bladder cancer cell invasion and migration.

### miR-616-5p Promotes Bladder Cancer Cell Invasion and Migration *Via* Downregulating NR2C2 Expression

**Figure 4:**
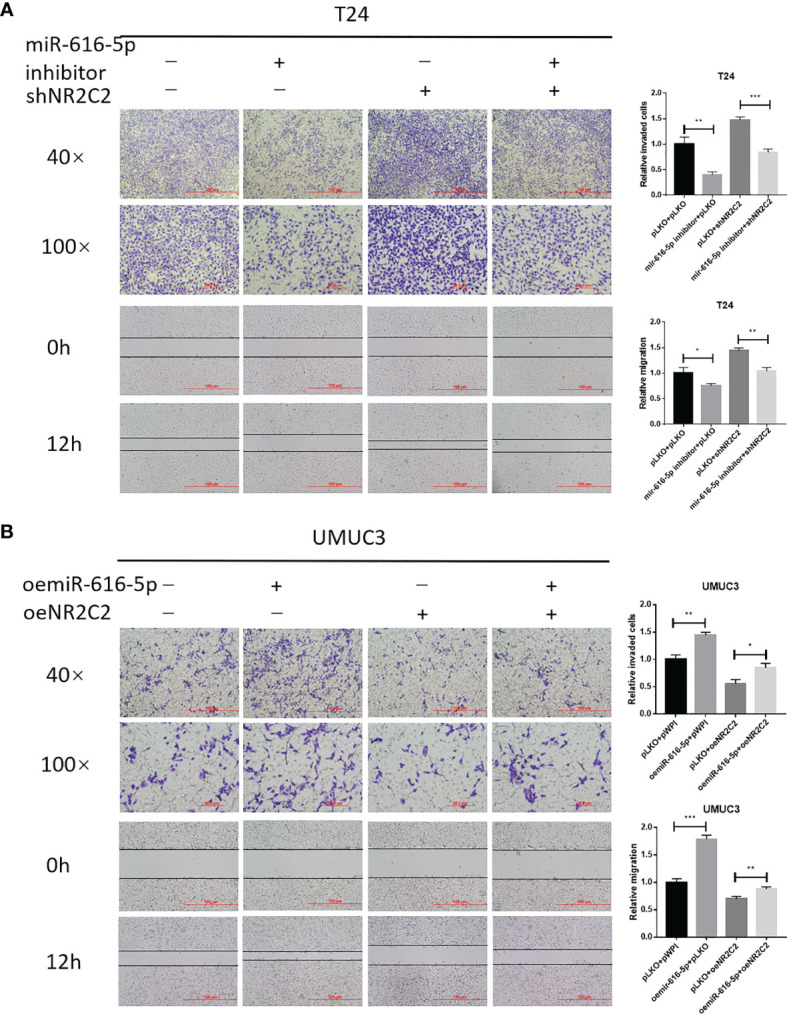
miR-616-5p promotes bladder cancer cell invasion and migration *via* downregulating the NR2C2 expression. **(A)** Chamber–transwell invasion and wound healing migration assays performed in T24 cells transduced with pLKO + pLKO, miR-616-5p inhibitor + pLKO, pLKO + shNR2C2, and miR-616-5p inhibitor + shNR2C2. **(B)** Chamber–transwell invasion and wound healing migration assays performed in UMUC3 cells transduced with pWPI + pWPI, oemiR-616-5p + pWPI, pLKO + oeNR2C2, and oemiR-616-5p + oeNR2C2. All quantification data are presented as mean ± SD, and significant differences are indicated by *P < 0.05, **P < 0.01, and ***P < 0.001 compared to the controls.

As shown in **Figures 2B**, **3D**, we found that miR-616-5p could increase the invasion and migration of bladder cancer, but NR2C2 could decrease the invasion and migration of bladder cancer. In this part, we performed a rescue experiment to check the relationship between miR-616-5p and NR2C2 on the phenotype of bladder cancer. When we knocked down miR-616-5p and NR2C2 at the same time, the invasion and migration reduction caused by the miR-616-5p inhibitor was partially reversed by shNR2C2 in T24 cells (**Figure 4A**). Similarly, oeNR2C2 could partially reverse the oemiR-616-5p-enhanced invasion and migration in UMUC3 cells (**Figure 4B**). To sum up, **Figure 4** illustrates that miR-616-5p could promote invasion and migration *via* altering the NR2C2 expression in multiple bladder cancer cell lines.

### Mechanism Dissection on How miR-616-5p Alters NR2C2 Expression

**Figure 5:**
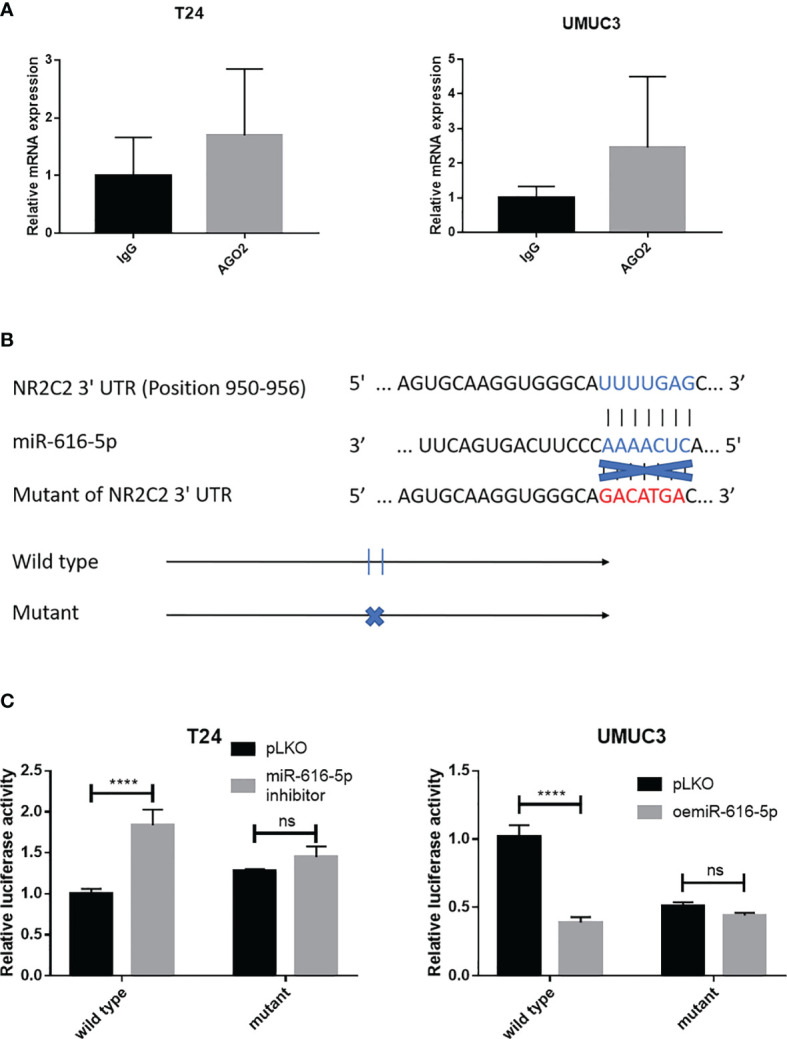
Mechanism dissection on how miR-616-5p alters NR2C2 expression: *via* binding to the 3′UTR of NR2C2. **(A)** RNA binding protein immunoprecipitation assay for miRNAs using IgG or AGO2 antibody in T24 cells and UMUC3 cells. The qRT-PCR assay was used to detect NR2C2 expression in each group. **(B)** Sequence alignment of NR2C2 3′UTR with wild-type *versus* mutant potential miR-616-5p targeting sites. The wild-type and mutant psiCHECK2- NR2C2 3′UTR reporter constructs. **(C)** Luciferase reporter activity after transfection of the wild-type or mutant NR2C2 3′UTR reporter construct in T24 cells transduced with pLKO and miR-616-5p inhibitor and UMUC3 cells transduced with pLKO and oemiR-616-5p. All quantification data are presented as mean ± SD, and significant differences are indicated by and ****P < 0.0001 and ns, not significant compared to the controls.

To check whether miRNAs could bind to NR2C2 mRNA, RNA binding protein immunoprecipitation assay was performed in T24 and UMUC3 cells. The results showed that the NR2C2 expression in the AGO2 group was higher than that in the IgG group (**Figure 5A**). To verify how miR-616-5p can regulate NR2C2 expression at the molecular level, we identified some potential binding sites located on the 3′UTR of NR2C2-mRNA (http://www.targetscan.org). We then applied the reporter assay with the psicheck2 vector carrying the wild-type and mutant miRNA target sites (**Figure 5B**). The luciferase assay results revealed that miR-616-5p inhibitor could increase the luciferase activity in the wild-type group, while it had little effect in the mutant group. On the other hand, oemiR-616-5p could attenuate the luciferase activity in the wild-type group, with little effect in the mutant group (**Figure 5C**). Thus, **Figure 5** indicated that miR-616-5p could suppress the expression of NR2C2 protein *via* directly binding to the 3′UTR region of NR2C2 mRNA.

### NR2C2 Could Act as a Tumor-Suppressor Gene in Bladder Cancer

**Figure 6:**
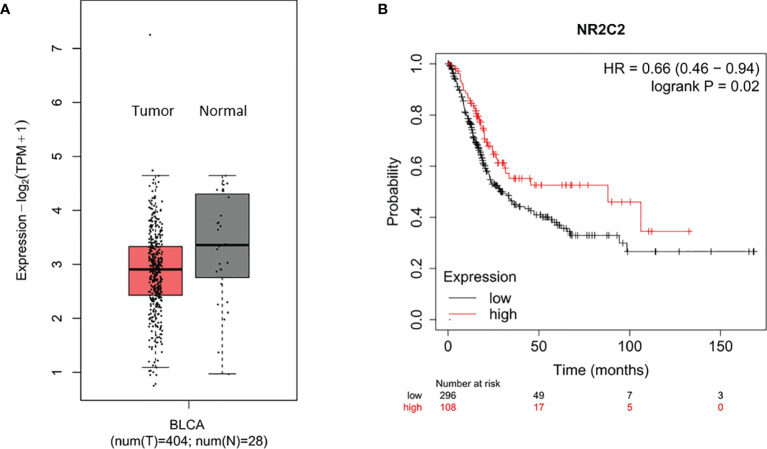
NR2C2 could act as a tumor-suppressor gene in bladder cancer based on TCGA and GTEx database. **(A)** Expression of miR-616-5p in bladder cancer tissues and adjacent normal tissues based on The Cancer Genome Atlas and GTEx databases. **(B)** Overall survival curve shows that a higher NR2C2 expression correlates with better prognosis in bladder cancer patients.

To ascertain the clinical significance of NR2C2 in bladder cancer. Based on TCGA and GTEx database, we found that the expression of NR2C2 in bladder cancer tissues was lower than that in adjacent normal tissues (**Figure 6A**) (20). Then, we analyzed the prognostic data of bladder cancer patients based on the TCGA database (21). The overall survival curve shows that the prognosis of patients with bladder cancer is positively associated with the NR2C2 expression (**Figure 6B**). The clinical data further verified our *in vitro* results that NR2C2 could act as a tumor suppressor for bladder cancer.

## Discussion

Metastasis is an important factor affecting the prognosis of patients with cancer. In metastatic bladder cancer patients, the 5-year overall survival rate is only about 15% (7). Therefore, it is essential to understand the underlying mechanism of bladder cancer metastasis. Specifically, studying the molecular biological process of bladder cancer development may provide promising biomarkers and potential therapeutic targets to prevent tumor metastasis and improve the survival rate. miRNAs have been revealed to regulate invasion and migration to facilitate tumor metastasis in various cancers (22). In our study, we found that miR-616-5p was upregulated both in bladder cancer tissues and in cells. miR-616-5p could promote bladder cancer invasion and migration *via* downregulating the NR2C2 expression. Moreover, prognostic data based on TCGA database consistently revealed that NR2C2 could act as a tumor suppressor in bladder cancer.

miR-616 is a recently found miRNA that is associated with cancer genesis and progress. miR-616 includes two mature miRNAs: miR-616-5p and miR-616-3p. Previous studies reported that miR-616 could promote the progress of several cancers, including breast cancer, non-small cell lung cancer, pancreatic carcinoma, and ovarian cancer (23–26). The limitation of these articles is that they did not accurately define which mature miRNA of miR-616. To the best of our knowledge, only three publications focused on the function of miR-616-5p in cancer progress (12, 13, 27). Previous studies reported that miR-616-5p could promote both ovarian cancer and lung cancer *via* targeting the GSK3 beta pathway (12, 13). In the study of Zhang, decreased miR-616-5p could promote cell viability and inhibit apoptosis *via* downregulating the DUSP2 expression in gastric cancer (27). There is no report on the function of miR-616-5p in bladder cancer yet. We first discovered that miR-616-5p had the function of promoting bladder cancer progress. Our findings indicate the important role of miR-616-5p in the fundamental processes of bladder cancer metastasis.

The main function of miRNA is to bind to the 3′UTR of target mRNAs, thus leading to the degradation of mRNAs (28). In order to find out the downstream target of miR-616-5p, we conducted Western blot assay to screen several tumor-related genes which could act as tumor suppressors in cancers (29–34). We found that NR2C2 could be a potential downstream target, and we did the invasion and migration assays to verify its function in bladder cancer. Indeed NR2C2 has been reported to be a tumor-related gene in genitourinary cancers, including prostate cancer and renal cell carcinoma, but with no report on bladder cancer yet (16, 17, 35). In prostate cancer, NR2C2 could prevent or delay prostate tumorigenesis *via* regulating the ATM expression at the transcriptional level and could suppress prostate cancer invasion *via* altering the TIMP-1/MMP2/MMP9 signals (17, 35). In renal cell carcinoma, NR2C2 could promote renal cell carcinoma metastasis *via* HGF/Met and miR490-3p/vimentin signals (16, 36). In our study, we revealed the role of NR2C2 as a tumor suppressor in bladder cancer. Our findings provide further evidence for the regulation between NR2C2 and genitourinary cancers.

Even though we first found that miR-616-5p could promote bladder cancer invasion and migration *via* depressing the NR2C2 expression, there are some limitations in this study. First, our study focused on *in vitro* experiments and clinical data analysis; an *in vivo* verification experiment may need to be conducted. Second, the potential downstream targets of NR2C2 may need to be identified. Despite these limitations, the authors understand that the findings of this study may provide promising evidence for the molecular mechanisms involved in the progression and development of bladder cancer.

In conclusion, our study investigated the potential role of miR-616-5p in bladder cancer progression and its underlying mechanisms. We provided the first evidence that upregulated miR-616-5p could promote bladder cancer invasion and migration by targeting NR2C2. Therefore, our results suggest that the miR-616-5p/NR2C2 pathway could be a potential therapeutic target for bladder cancer.

## Data Availability Statement

The datasets presented in this study can be found in online repositories. The names of the repository/repositories and accession number(s) can be found in the article/supplementary material.

## Author Contributions

BF and XZ designed the study, WR conducted the experiment under the guidance of JC and JD, and JH and HL analyzed the clinical data. All authors contributed to the article and approved the submitted version.

## Funding

Funding for this work was provided by the Natural Science Foundation of Hunan Province (nos. 2019JJ40488 and 2018JJ2623) and the National Natural Science Foundation of China (nos. 81873626, 81902592, and 82070785).

## Conflict of Interest

The authors declare that the research was conducted in the absence of any commercial or financial relationships that could be construed as a potential conflict of interest.

## Publisher’s Note

All claims expressed in this article are solely those of the authors and do not necessarily represent those of their affiliated organizations, or those of the publisher, the editors and the reviewers. Any product that may be evaluated in this article, or claim that may be made by its manufacturer, is not guaranteed or endorsed by the publisher.
